# DNA methylation profiling demonstrates superior diagnostic classification to RNA-sequencing in a case of metastatic meningioma

**DOI:** 10.1186/s40478-020-00952-3

**Published:** 2020-06-09

**Authors:** Harish N. Vasudevan, Maria R. H. Castro, Julieann C. Lee, Javier E. Villanueva-Meyer, Nancy Ann Oberheim Bush, Michael W. McDermott, David A. Solomon, Arie Perry, Stephen T. Magill, David R. Raleigh

**Affiliations:** 1 Department of Radiation Oncology, University of California San Francisco, California, 94143 USA; 2 Department of Neurological Surgery, University of California San Francisco, California, 94143 USA; 3 Department of Pathology, University of California San Francisco, California, 94143 USA; 4 Department of Radiology and Biomedical Imaging, University of California San Francisco, California, 94143 USA

**Keywords:** Meningioma, Metastasis, DNA methylation, RNA-seq, RNA sequencing, Case report

## Abstract

Meningiomas are the most common primary intracranial tumors, but meningioma metastases are rare. Accordingly, the clinical workup, diagnostic testing, and molecular classification of metastatic meningioma is incompletely understood. Here, we present a case report of multiply recurrent meningioma complicated by liver metastasis. We discuss the patient presentation, imaging findings, and conventional histopathologic characterization of both the intracranial lesion and the metastatic focus. Further, we perform multiplatform molecular profiling, comprised of DNA methylation arrays and RNA-sequencing, of six stereotactically-guided samples from the intracranial meningioma and a single ultrasound-guided liver metastasis biopsy. Our results show that DNA methylation clusters distinguish the liver metastasis from the intracranial meningioma samples, and identify a small focus of hepatocyte contamination with the liver biopsy. Nonetheless, DNA methylation-based classification accurately identifies the liver metastasis as a meningioma with high confidence. We also find that clustering of RNA-sequencing results distinguishes the liver metastasis from the intracranial meningiomas samples, but that differential gene expression classification is confounded by hepatocyte-specific gene expression programs in the liver metastasis. In sum, this case report sheds light on the comparative biology of intracranial and metastatic meningioma. Furthermore, our results support methylation-based classification as a robust method of diagnosing metastatic lesions, underscore the broad utility of DNA methylation array profiling in diagnostic pathology, and caution against the routine use of bulk RNA-sequencing for identifying tumor signatures in heterogeneous metastatic lesions.

## Introduction

Meningiomas metastases are rare, occurring in less than 1% of all cases [1–3]. However, the rate of metastasis increases to 2% for World Health Organization (WHO) grade II meningiomas, and is nearly 9% for WHO grade III meningiomas [4], most frequently in the lungs, liver, lymph nodes, or bone [3, 5, 6]. Recent studies have shed light on the genomic, epigenomic, and transcriptomic signatures of intracranial meningiomas [7–10], but little is known about the molecular features underlying meningioma metastases. To date, there is only one case report evaluating the genomic profile of metastatic meningioma, revealing a single dominant clone in both the primary and metastatic tumors [11]. This study was limited to whole exome sequencing (WES), which, in contrast to DNA methylation profiling and RNA-sequencing (RNA-seq), cannot stratify the vast majority of meningiomas according to clinical outcomes [8, 9]. Here, we report a case of multiply recurrent metastatic meningioma presenting with simultaneous intracranial and hepatic progression. The metastasis was biopsied for diagnostic purposes just prior to craniotomy for resection of the intracranial tumor, presenting a unique opportunity to investigate the molecular features of matched primary and metastatic meningioma.

## Case presentation

**Fig. 1:**
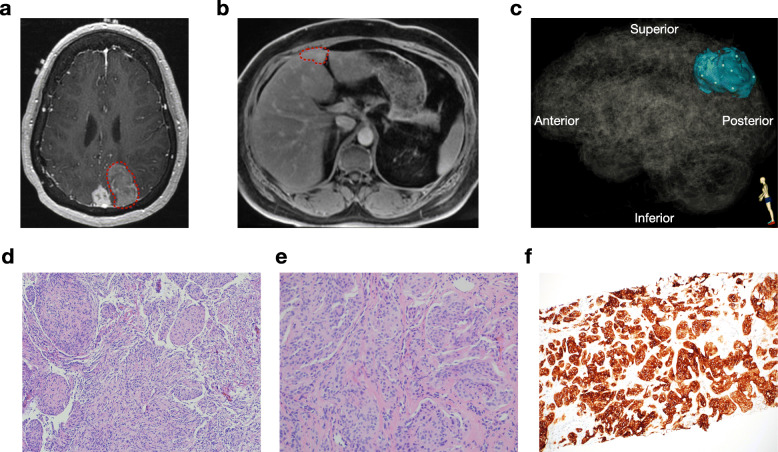
Pre-operative magnetic resonance imaging and histopathology of intracranial and metastatic meningioma. **a** Post-contrast T1 axial MR images show the enlarging left parieto-occipital meningioma (red circle), and a stable parasagittal meningioma**. b** Post-contrast liver MRI shows a metastatic lesion in segment IVb (red circle). **c** Three-dimensional stereotactic meningioma sampling map for 6 intracranial meningioma samples reconstructed from preoperative magnetic resonance imaging. **d** H&E stain of the intracranial meningioma sample (10x). **e** H&E stain of the liver metastasis core biopsy (20x). **f** SSTR2A immunohistochemistry of the liver metastasis core biopsy (10x)

The patient is a 52-year-old female who initially underwent resection of a sporadic intracranial meningioma, WHO grade I, at age 27. At age 38, she underwent salvage resection for multifocal intracranial recurrence, with surgical pathology revealing transformation to atypical meningioma, WHO grade II. At age 48, she again presented with intracranial progression and was treated with external beam radiotherapy, followed by stereotactic radiosurgery to satellite intracranial lesions at age 50. At age 52, a surveillance MRI of the brain revealed further intracranial recurrence (Fig. 1a), and she underwent whole body imaging that identified a liver metastasis (Fig. 1b). Subsequent salvage resection (Fig. 1c) with concurrent Cs-131 brachytherapy of the growing intracranial tumor, and ultrasound-guided liver biopsy, again demonstrated intracranial atypical meningioma, WHO grade II (Fig. 1d), as well as metastatic meningioma in the liver (Fig. 1e), which was verified using immunohistochemistry for somatostatin receptor type 2a (Fig. 1f) [12].

**Fig. 2:**
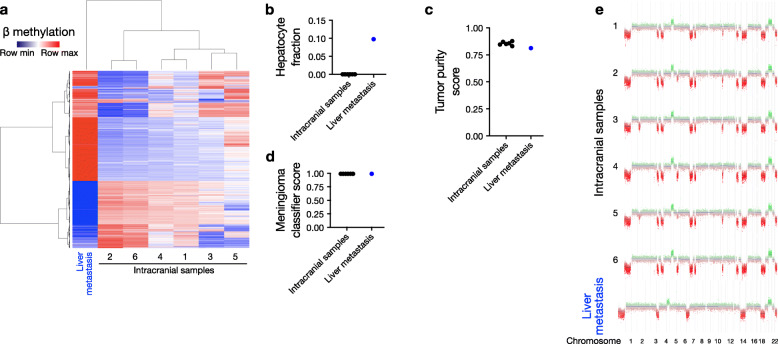
DNA methylation analysis reveals minimal contamination, conserved epigenetic classification, and concordant copy number variants in meningioma liver metastasis. **a** Hierarchical clustering of the top 2000 most variable DNA methylation probes segregates the liver metastasis from the intracranial meningioma samples. **b** Cell type deconvolution demonstrates ~ 10% hepatocyte composition within the liver metastasis, which is not identified in the intracranial samples. **c** Tumor purity analysis reveals no significant difference between intracranial and liver metastasis samples. **d** Random forest classification correctly identifies all six intracranial sites and the liver metastasis as meningioma with high confidence (classifier score = 0.99 for all samples). **e** Copy number variant (CNV) profiles show no significant private alterations in the liver metastasis compared to intracranial samples

To elucidate the molecular features associated with the meningioma metastasis, we performed DNA methylation profiling and RNA-seq on 6 spatially distinct sites from the intracranial meningioma and the liver metastasis. Intracranial samples, as well as the liver core biopsy, were flash frozen in liquid nitrogen immediately after collection, and DNA and RNA were simultaneously extracted from the same sample from each site. Unsupervised hierarchical clustering of methylation data revealed that the liver metastasis demonstrated a distinct epigenetic profile from the 6 intracranial lesions (Fig. 2a), likely resulting from hepatocellular contamination in the metastatic sample. In support of this hypothesis, deconvolution of cell types from methylation data [13], with a focus on hepatocytes, showed a small hepatocyte fraction exclusively in the liver metastasis (0% versus 9.8%, Fig. 2b). However, tumor purity analysis from methylation data [15] demonstrated similar percentages in the 6 intracranial samples compared to the liver metastasis (83–88% versus 81%, Fig. 2c). Consistently, all 7 samples demonstrated high concordance with meningioma methylation profiles based on tumor classification via a random forest model (99% versus 99%, Fig. 2d) [14], suggesting that the biopsied liver lesion was indeed primarily composed of metastatic meningioma cells, rather than contaminating stromal cells or infiltrated hepatocytes. Moreover, when we calculated copy number variants (CNVs) based on DNA methylation profiles [16], we found no private CNVs in the liver metastasis compared to the intracranial samples (Fig. 2e), and that all samples demonstrated loss of chromosome 22q, which harbors the meningioma tumor suppressor gene *NF2*. However, we did observe 4 CNVs that were present in the intracranial lesions but lost in the liver metastasis, which may have been driven, in part, by the underlying normal hepatocyte contamination in the metastatic sample. Notably, these changes did not appear to affect DNA methylation-based tumor classification, and could, alternatively, have been reflective of metastasis of a meningioma clone not captured in the 6 intracranial meningioma samples we profiled. In summary, DNA methylation profiling indicates that metastatic meningioma, while containing detectable contaminating cells, is primarily composed of meningioma cells with a similar CNV profile to matched intracranial samples.

**Fig. 3:**
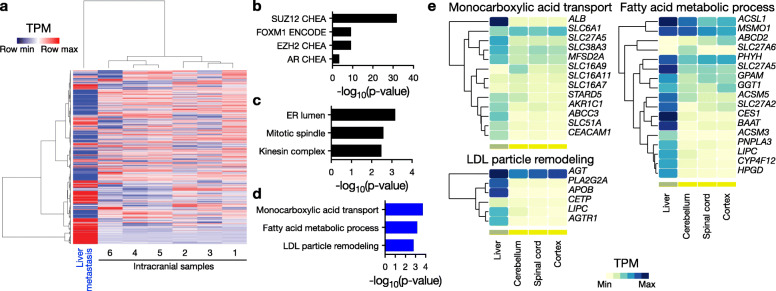
RNA-sequencing analysis demonstrates that native hepatocytes drive the transcriptomic signature of meningioma liver metastasis. **a** Hierarchical clustering of the top 2000 most variable genes segregates the meningioma liver metastasis from the intracranial samples. **b, c** Gene ontology analysis of increased transcripts in intracranial meningioma samples shows (b) enrichment for SUZ12 and FOXM1 transcription factor pathways and (c) mitotic spindle function. **d** Gene ontology analysis of increased transcripts in the meningioma liver metastasis shows enrichment for liver specific processes, such as monocarboxylic acid transport, fatty acid processing, and LDL remodeling. **e** Analysis of tissue specific expression patterns demonstrates that gene sets enriched in the meningioma liver metastasis have liver-specific expression patterns

We next used RNA-seq and differential expression analysis to compare the transcriptomes of the 6 intracranial samples with the liver metastasis. Unsupervised hierarchical clustering of transcriptomic data segregated the liver metastasis from the intracranial samples (Fig. 3a). Notably, a large number of genes were detected exclusively in the intracranial or metastatic samples, consistent with contaminating non-meningioma cells. In order to minimize contaminating hepatocyte signatures, we filtered RNA-seq data to identify only those genes expressed at a transcripts per million (TPM) level greater than 1 in all 7 samples, resulting in a total of 16,513 genes (45% of the initial gene list). We then selected genes with a log2 fold change greater than 2, which resulted in 628 enriched genes in the intracranial meningioma samples, and 726 enriched genes in the metastasis (Supplementary Table 1). Gene ontology analysis revealed enrichment of SUZ12 and FOXM1 transcription factor networks (Fig. 3b) and mitotic spindle function (Fig. 3c) in the intracranial meningioma samples, consistent with the established roles of these pathways in regulating meningioma cell proliferation [9]. In contrast, genes enriched in the liver metastasis showed overrepresentation of metabolic pathways, and SUZ12 and hepatocyte nuclear factor 4a (HNF4A) transcription factor networks, suggestive of liver-enriched gene expression programs, rather than metastatic meningioma (Fig. 3d). In support of this hypothesis, analysis of the tissue specific expression patterns of the metastasis gene set revealed enrichment of liver restricted transcripts (Fig. 3e). These data are consistent with the notion that bulk RNA-seq has limited utility for identifying molecular signatures in meningioma metastases, even with stringent filters from a relatively pure biopsy, as evidenced by pathology (Fig. 1e), histology (Fig. 1f), cell type deconvolution (Fig. 2b), tumor purity analysis (Fig. 2c), random forest tumor classification (Fig. 2d), and copy number variants (Fig. 2e).

## Conclusion and discussion

In summary, we performed DNA methylation profiling and RNA-seq of intracranial and metastatic samples from a recurrent meningioma. We found that both DNA methylation and RNA-seq distinguished the metastasis from intracranial meningioma samples, but while DNA methylation-based classification correctly identified the metastatic sample as meningioma in origin, RNA-seq of the same metastatic sample was confounded by hepatocyte contamination, even though the vast majority of the sample was comprised of meningioma cells. More broadly, these data further support the diagnostic utility of epigenetic profiling for meningioma, and suggest that DNA methylation-based classification of central nervous system tumors may be a robust assay for identifying metastatic lesions. However, these data also caution against RNA-seq based analysis of heterogeneous metastatic lesions, perhaps foreshadowing the potential importance of single cell approaches to elucidate the molecular mechanisms underlying metastasis. Finally, our findings underscore the importance of classical diagnostic features, such as histopathology and immunohistochemistry, for clearly establishing the diagnosis of metastatic meningioma when emerging technologies may be limited in ways that have yet to be rigorously defined, as is illustrated by this case.

## Methods

### Intracranial and metastatic meningioma sample collection

This study was approved by the authors institutional review board (IRB# 17–23,196). The patient provided written informed consent for research on both the liver and brain tumor samples (IRB #10–01318). We stereotactically collected 6 spatially distinct samples from the intracranial meningioma (from within the bulk of the tumor, away from the periphery at the brain/tumor interface, to minimize contamination from brain parenchyma cells) during craniotomy for tumor resection, and 1 sample from the liver metastasis via ultrasound-guided fine needle aspiration, for histopathology, DNA methylation profiling, and RNA-sequencing.

### Nucleic acid extraction for bulk RNA sequencing and DNA methylation profiling

DNA and RNA were isolated from the same biopsy specimen for each sample using the All-Prep Universal Kit (QIAGEN, Valencia, CA). Flash frozen tumor samples were thawed in RLT Plus Buffer with beta-mercaptoethanol and were mechanically lysed using a TissueLyzer (QIAGEN) with stainless steel beads at 30 Hz for 90 s. QiaCubes were used for standardized automated nucleic acid extraction per the manufacturer’s protocol (QIAGEN). RNA quality was assessed by chip-based electrophoresis (Agilent Technologies, Waldbronn, Germany), and clean-up was performed as needed using the RNeasy kit (QIAGEN). DNA quality was assessed by spectrophotometry, and clean-up was performed as needed using DNA precipitation.

### DNA methylation arrays and analysis

Methylation analysis was performed according to the manufacturer’s instructions on the Illumina Methylation EPIC Beadchip. Preprocessing and normalization were performed in R using the minfi Bioconductor package [17, 18]. Only probes with detection *P* < 0.05 in all samples were included for further analysis. Data were normalized using functional normalization [18]. Probes were filtered based on the following criteria: (i) removal of probes targeting the X and Y chromosomes, (ii) removal of probes containing a common single nucleotide polymorphism (SNP) within the targeted CpG site or on an adjacent basepair, and (iii) removal of probes not mapping uniquely to the hg19 human reference genome. Heatmaps were generated with custom code in R. Methylation based brain tumor classification [14], CNV estimation [14], and tumor purity analysis [15] were carried out as previously described.

### RNA-sequencing and analysis

Library preparation was performed using the TruSeq RNA Library Prep Kit v2 (RS-122- 2001, Illumina, San Diego, CA) and 50 bp single end reads were sequenced on an Illumina HiSeq 2500 at the Center for Advanced Technology at the University of California San Francisco. Quality control of FASTQ files was performed with FASTQC, and after trimming of adapter sequences, reads were further filtered to remove bases that did not have an average quality score of 20 within a sliding window across 4 bases (http://www.bioinformatics.babraham.ac.uk/projects/fastqc/). Reads were subsequently mapped to the human reference genome hg19 using HISAT2 with default parameters [19]. Transcript abundance estimation in transcripts per million (TPM) were performed using DESeq2 [20]. Heatmaps were generated with custom code in R and normalized by row expression values. Given our inclusion of a single liver metastasis, we used a combined absolute expression cutoff and fold change threshold approach to identify differentially expressed genes between intracranial and metastatic samples. We identified differentially expressed transcripts with an expression cutoff (TPM > 1) and fold change threshold (| log2FC | > 2). Gene ontology analysis was carried out in ENRICHR [9, 21]. Tissue specific expression of transcripts was obtained from the Genotype-Tissue Expression (GTEx) Project, which is supported by the Common Fund of the Office of the Director of the National Institutes of Health, and by NCI, NHGRI, NHLBI, NIDA, NIMH, and NINDS. The data used for the analyses described in this manuscript were obtained from the GTEx Portal on 04/03/20.

## Supplementary information

**Additional file 1: Supplementary Table 1.** Multiplatform molecular profiling. List of all genes detected at transcripts per million (TPM) > 1 (“Filtered_TPM > 1”) and genes enriched in the liver metastasis (“Up_in_Met”) or intracranial samples (“Down_in_Met”) based on combined absolute expression and relative fold change thresholds.

## Data Availability

Processed gene expression data for all samples are available in Supplementary Table 1. Raw data have been deposited to GEO and SRA. DNA methylation data was deposited in GEO: GSE151067.
